# Extracting *in Situ* Charge Carrier Diffusion Parameters in Perovskite Solar Cells with Light Modulated Techniques

**DOI:** 10.1021/acsenergylett.1c00871

**Published:** 2021-05-24

**Authors:** Agustín Bou, Haralds A̅boliņš, Arjun Ashoka, Héctor Cruanyes, Antonio Guerrero, Felix Deschler, Juan Bisquert

**Affiliations:** † Institute of Advanced Materials (INAM), Universitat Jaume I, Avda. Sos Baynat sn, 12006 Castelló, Spain; ‡ Cavendish Laboratory, University of Cambridge, J.J. Thomson Avenue, CB3 0HE, Cambridge, United Kingdom; § Walter-Schottky-Institute, Physics Department, Technical University Munich, Am Coulombwall 4, Garching bei München, Germany

## Abstract

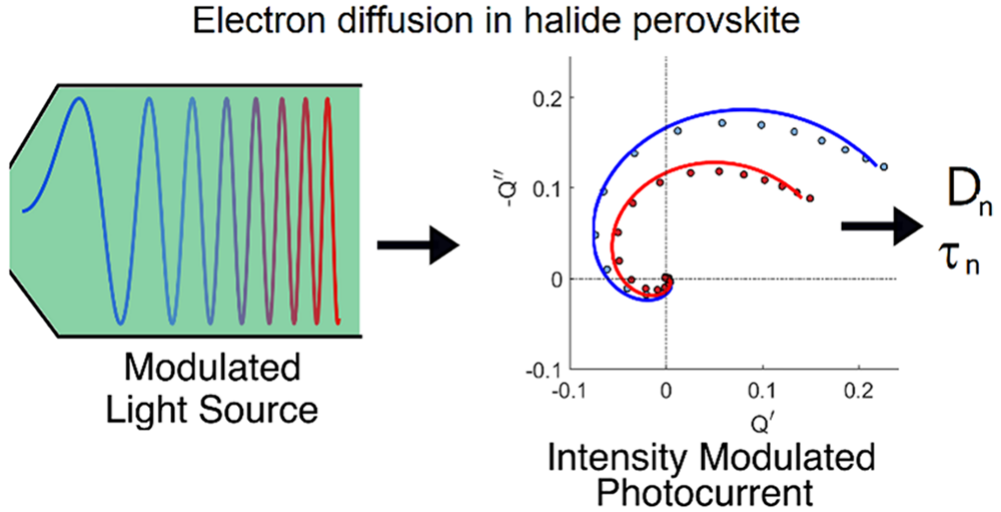

Frequency resolved methods are widely used to determine device properties of perovskite solar cells. However, obtaining the electronic parameters for diffusion and recombination by impedance spectroscopy has been so far elusive, since the measured spectra do not present the diffusion of electrons. Here we show that intensity modulated photocurrent spectroscopy (IMPS) displays a high frequency spiraling feature determined by the diffusion-recombination constants, under conditions of generation of carriers far from the collecting contact. We present models and experiments in two different configurations: the standard sandwich-contacts solar cell device and the quasi-interdigitated back-contact (QIBC) device for lateral long-range diffusion. The results of the measurements produce the hole diffusion coefficient of *D*_*p*_ = 0.029 cm^2^/s and lifetime of τ_*p*_ = 16 μs for one cell and *D*_*p*_ = 0.76 cm^2^/s and τ_*p*_ = 1.6 μs for the other. The analysis in the frequency domain is effective to separate the carrier diffusion (at high frequency) from the ionic contact phenomena at a low frequency. This result opens the way for a systematic determination of transport and recombination features in a variety of operando conditions.

Metal halide perovskites (MHP) have raised enormous research efforts as a future high efficiency low-cost photovoltaic platform and also for various semiconductor electronics and photonics applications. Consequently, a priority of current research is the characterization of electronic parameters such as the electron diffusion coefficient, *D*_*n*_, and the electron recombination lifetime, τ_*n*_. There have been presented a large number of evaluations of the diffusion length *L*_*n*_ = (*D*_*n*_τ_*n*_)^1/2^ measured by time transient methods in the archetype perovskite solar cells (PSC) with a methylammonium (MA) cation, namely, MAPbI_3_ and MAPbBr_3_.^1,2^ There have also been abundant determinations of carrier mobilities by a range of techniques: space-charge limited-current (SCLC), Hall effect, THz frequency measurements, etc. The results span a variety of values from *D*_*n*_ = 0.01 cm^2^ s^–1^ to 4 cm^2^ s^–1^.^3,4^

In this Letter, we address the observation of electronic diffusion characteristics in the framework of small perturbation frequency modulated techniques that allow the study of the full device operation in a wide range of scales from very low millihertz frequencies to megahertz phenomena. These methods yield us the important advantage that they can be applied in full efficient devices (in contrast to contactless methods such as THz spectroscopy) avoiding effects of ionic polarization that plague other techniques, as it has been well described recently,^5^ provided that the diffusion effect is observed at high frequency, far from the low frequency ionic polarization. Therefore, it is important to find the spectral signatures of diffusion in PSCs.

As a reference, the diffusion of electrons was distinctly observed by impedance spectroscopy (IS)^6^ and intensity modulated photocurrent spectroscopy,^7^ and these became dominant methods of analysis in dye solar cells. The diffusion effect is usually manifested as a 45° inclined line at high frequency in the complex plane representation of the spectra. This is the Warburg impedance with the square root dependence on the angular frequency as *Z*(ω) ∝ (*i*ω)^−1/2^, clearly indicating the presence of a diffusion transport resistance.^8^ An enormous number of papers have analyzed the IS response of PSCs, and such a response has not been observed. The usual reason to explain the absence of such an observation is that the transport resistance it too small due to the large electron mobility/diffusion coefficient and becomes absorbed in the series resistance. Observations of low frequency Warburg elements in IS studies^9,10^ have been attributed to ionic diffusion. There have been also a significant number of studies of MHP using IMPS,^11−16^ but the spectral observation of Warburg features has not been achieved.

Often, the IMPS transfer function in MHP shows the curious feature that the spectra turn to real negative values (second quadrant) at high frequency, as indicated in Figure 1. (It is different from the negative value observed at *very low frequency*.^17−19^) The high frequency feature has been often explained in the literature as the effect of RC attenuation,^7,11,14^ that is, the large frequency negative feature is associated with the impedance of series and parallel elements in addition to diffusion. This type of effect is obviously uninteresting for the observation of diffusion. In any case, it provides a correction of the spectra by the impedance elements that can be measured independently. However, another effect associates a negative spiraling IMPS feature with the photocurrent created by carriers generated far from the collecting contact.^20,21^ The observations of Figure 1 indicate an opportunity where the diffusion-recombination effect dominates the frequency response of a PSC and enables the determination of the physical parameters. These experimental responses have been recently observed in a systematic fashion and related to negative transient photocurrent spikes.^22^

**Figure 1:**
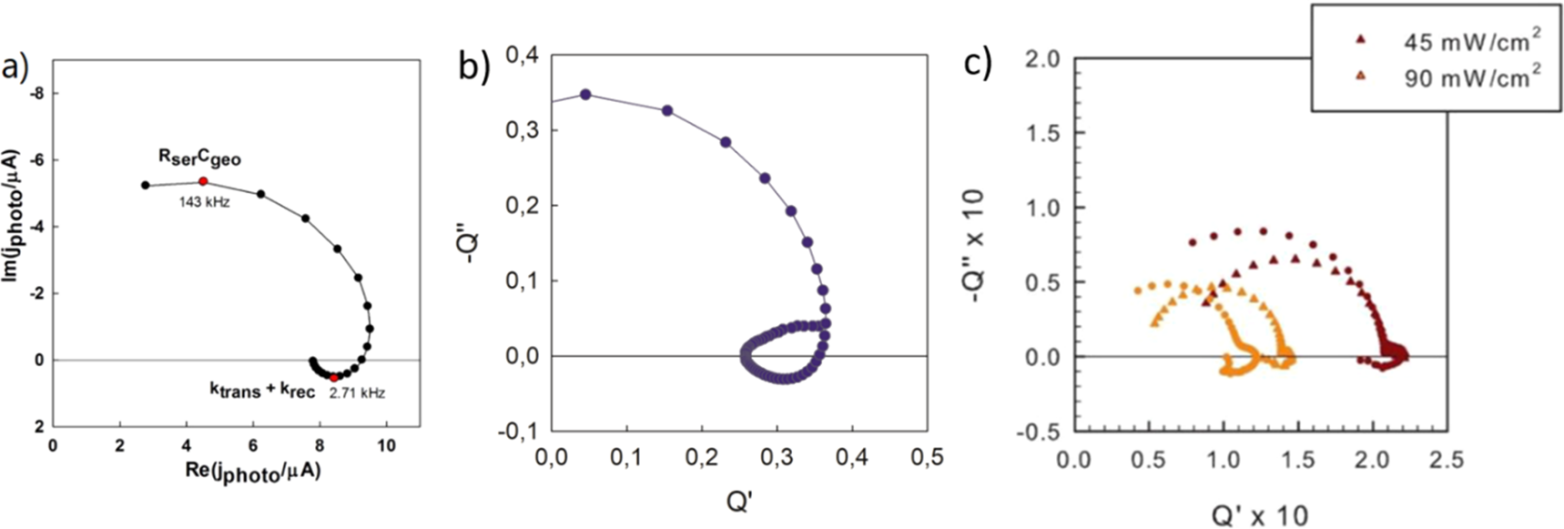
IMPS response for PSCs indicating an excursion to the second quadrant in the case of a and b and staying in the first quadrant for c. (a) Reproduced with permission from ref (11). Copyright 2015 American Chemical Society. (b) Reproduced with permission from (16). Copyright 2020 American Chemical Society. (c) Reproduced with permission from (12). Copyright 2019 American Chemical Society.

Interestingly, the excursion to the second quadrant in Figure 1b, in which the real part of the IMPS transfer function becomes negative, is found in a configuration with a large perovskite layer that provokes a nonuniform generation profile. In this case, the generation profile is that represented in Figure 2a, where the generation of one kind of carrier is localized far away from their collecting contact. This is not the case of Figure 1c, where the thin film configuration allows the electron–hole pair generation to take place throughout the entire perovskite layer, and then the IMPS real part remains positive up to high frequency.

**Figure 2:**
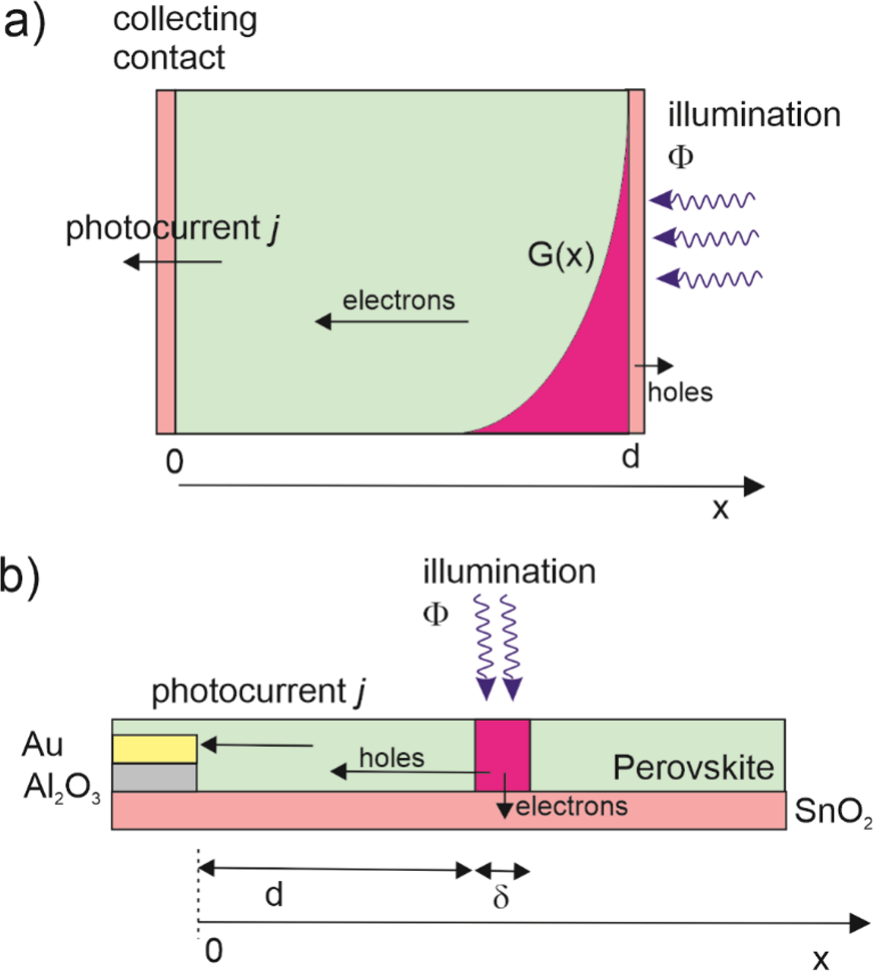
Scheme of the IMPS measurement for illumination far from the collecting contact. (a) The solar cell configuration. (b) Planar sample with lateral contact illuminated from above.

In this paper, we will carry out a systematic investigation of models and experiments on the high frequency negative loop in the diffusion-recombination systems applied to halide perovskite solar cells. We derive the model for two independent experimental configurations, indicated in Figure 2: the standard sandwich solar cell for diffusion perpendicular to the electrodes, Figure 2a, and a quasi-interdigitated back-contact (QIBC) for lateral diffusion, Figure 2b.^23^ This last structure has also been used by multiple groups for the fabrication of all back contacted solar cells with efficiencies up to 11.2%.^24−30^ A number of papers have studied lateral diffusion of electronic carriers in perovskites, using time transient methods.^31−33^ Excitation frequency-dependent photocurrent studies on QIBC devices have, however, never been performed, and could yield valuable insights on charge carrier dynamics for both a better understanding of intrinsic material and interface properties, as well as optimization of the back-contact structures for even higher efficiency solar cells. We show that electron diffusion coupled to recombination is clearly observed in both methods, and we provide a fitting method that allows us to extract the main parameters.

## Method 1. Diffusion between Parallel Contacts

The analysis of the IMPS spectra requires solving the system of the scheme of Figure 2a. Incident photon flux Φ arrives at the solar cell at *x* = *d*. The equation for the excess electron density *n* (over the equilibrium concentration *n*_0_) is
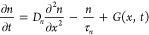
1where *G*(*x*) = αΦ exp(α(*x* – *d*)) is an exponential profile of generation dependent on the absorption coefficient α. The boundary conditions under short-circuit conditions are *n*(*x* = 0) = 0 and
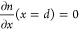
2

The photocurrent density *j*_*e*_ at the collecting contact at *x* = 0 is
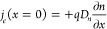
3

The IMPS transfer function *Q*(ω) = *Q*′(ω) + *iQ*″(ω) is obtained by the quotient of the small modulated input/output
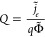
4

This problem is solved in ref (7), and a broad variety of illumination conditions are presented in ref (21). For the sake of completion, we describe the solution in the SI. The result is
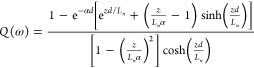
5where
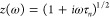
6

**Figure 3:**
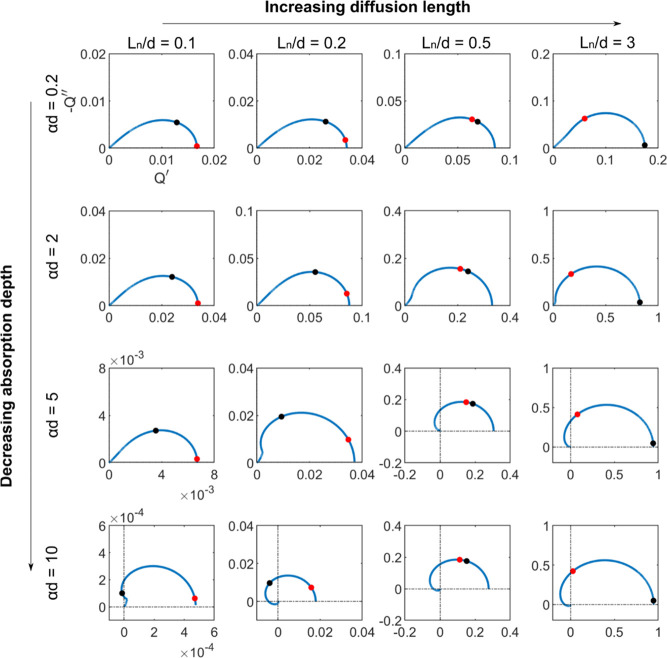
Complex plane plots of the IMPS transfer function for several relative values of light absorption distance and diffusion length. Rows are for equal absorption length and columns for equal diffusion length. Red points indicate the characteristic time constant for diffusion, ω_*g*_ = (π^2^/2)*D*_*n*_/*d*^2^, and the black ones are the characteristic time for recombination, ω_rec_ = 1/τ_*n*_. No RC attenuation is considered.

The shapes of the IMPS spectra generated by eq 5 depend on the light absorbance mode and the diffusion-recombination features of the material. The physical parameters for absorbance and extraction affecting the form of the spectra are the light absorption length, α^–1^, and the diffusion length, *L*_*n*_, respectively. These parameters transform the spectral shape depending on whether they are shorter or longer than the cell thickness *d*, and the different kind of spectra that are obtained are shown in Figure 3. In the spectra, we show the characteristic time constants^8^ for diffusion across the layer thickness
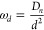
7and for recombination
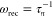
8

Note the proportions between characteristic distances and frequencies
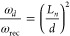
9

A Warburg-like spectral feature at high frequencies (*i*ω)^−1/2^ is obtained when the light is generated across the full thickness (α^–1^ > *d*), either for short or long *L*_*n*_ (top row of Figure 3). In the bottom rows, it is noted that looping spectra producing a negative *Q*′ at high frequency (NHF) appear only when the absorption length is much shorter than the cell thickness. Another required condition for this feature is the diffusion length being longer than the absorption. These conditions are expressed respectively as α^–1^ ≪ *d* and α^–1^ < *L*_*n*_. Then, loops appear in the α^–1^ < *d* < *L*_*n*_ case and also in the α^–1^ < *L*_*n*_ < *d* case. This analysis confirms that the NHF loop is associated with a collection of charges generated only far from the collecting contact. When the light is absorbed in a distance comparable to that of the cell (second row), no negative values of *Q*′ are obtained but the spectra turn from Warburg like into a semicircle as the *L*_*n*_ increases.

To better appreciate the analytical shape of the function of interest, we present in the Supporting Information some approximations that can be obtained when the diffusion length is longer that the cell thickness (*L*_*n*_ > *d*) and the light is completely absorbed in a short region. The high frequency limit is
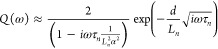
10

The spectral dependences of this function are shown in Figure SI.1. The complex exponential function that depends on  is the one that loops and spirals into the negative *Q*′ axis.

**Figure 4:**
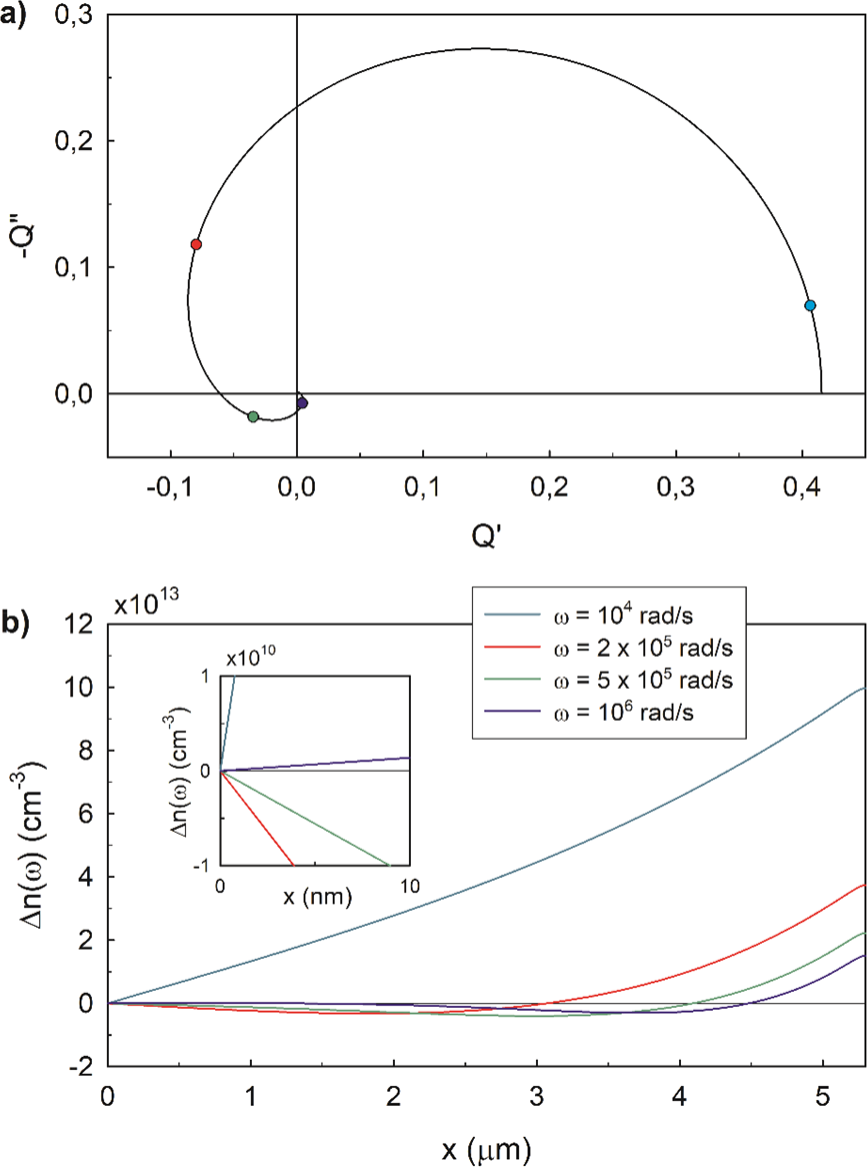
(a) IMPS complex plane plot for α*d* = 10 and *L*_*n*_/*d* = 1/2, indicating the angular frequencies for the concentration profiles in part b. (b) Plot of the spatial distribution of Δ*n* = *ñ*(ω, *x*), the excess charge carrier concentration induced by an ac flux perturbation of Φ̃ = 1.58 × 10^15^ s^–1^ cm^–2^, for an angular frequency representative of each quadrant, as indicated in part a; inset zooms the region close to the contact at *x* = 0.

In order to understand the negative values of the real part of the transfer function *Q*, we calculated the excess charge carrier concentration *ñ* along the cell associated with the small ac illumination; see the analytical expressions in the Supporting Information. We choose different representative regions of frequencies in the characteristic spectrum with the NHF loop, shown in Figure 4.

The excess carrier concentration profiles at the frequencies of different quadrants, as indicated in Figure 4a, are shown in Figure 4b. The extracted current is proportional to the gradient of carrier concentration at the contact. We observe that the frequencies in the first and fourth quadrants give a positive current, which agrees with a positive real value of *Q*′. In contrast, for frequencies in the second and third quadrants, the gradient is negative, meaning that we have a negative current and, thus, a negative real value of *Q*′. In summary, according to Figure 4, the negative *Q*′ occurs because the carriers generated at the contact decay rapidly at high frequency and make an upturn before the arrival to the collecting contact.

As mentioned earlier, it has been suggested in the literature that the additional impedances in the solar cell can produce a negative loop in the measured IMPS response, *Q*_meas_. The previous transfer function due to diffusion only is modified as
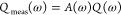
11

In the case of series resistance *R*_s_ and geometrical capacitance *C*_g_, the attenuation factor *A*(ω) is^7,21^
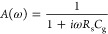
12

The RC attenuation can produce a considerable effect in high frequencies, as can be seen in Figure SI2. The RC attenuation with high RC values turns positive theoretical IMPS responses into the *Q*′ negative axis, as seen in Figure SI.2a. This effect makes the loop and Warburg IMPS responses undistinguished, whether light is completely absorbed or not. However, due to the fact that the RC values can be measured by the impedance spectroscopy technique, the RC attenuation can be controlled and removed to obtain the pure diffusion features.

**Figure 5:**
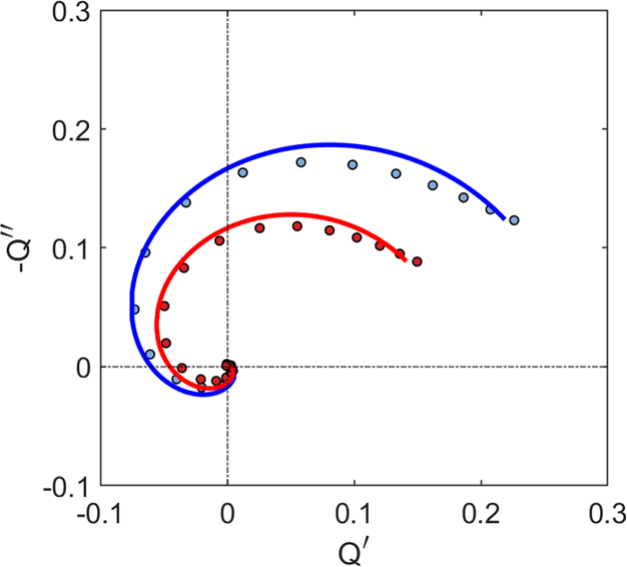
IMPS data (point) and fit (line) for a perovskite cell of 5.3 μm thickness illuminated with blue and red light, with an estimated absorption length of 40 and 140 nm, respectively.^34^ The series resistance is 10 Ω cm^2^, and the geometrical capacitance is 36 nF cm^–2^ as determined by impedance spectroscopy. The effect of RC attenuation is negligible.

In order to observe the diffusion-recombination parameters, we take the results of IMPS previously published^16^ for a mesoporous carbon-based perovskite solar cell of *d* = 5.3 μm illuminated with different wavelengths, related to the transport of holes. The data clearly show a large NHF loop feature as observed in Figure 5. The fit to eq 5 describes well the experimental data, including the spiraling feature to the origin at high frequency. We obtain values of τ_*p*_ = 16 μs for the lifetime and a diffusion coefficient of *D*_*p*_ = 0.029 cm^2^/s for the blue light fitting and values of τ_*p*_ = 20 μs and *D*_*p*_ = 0.034 cm^2^/s for red light, which are in agreement with the values for perovskite solar cells in the literature.^3^ These values produce diffusion lengths of *L*_*p*_ = 2.6 μm and *L*_*p*_ = 2.2 μm. These diffusion lengths match with the predictions from Figure 3, where we expected a diffusion length comparable to the length of the perovskite layer for looping spectra. As the value of the lifetime is much larger than the RC factor, the attenuation is not highly relevant in this case. It introduces a relative difference in the estimated values lower than 5% .

## Method 2. Lateral Diffusion

**Figure 6:**
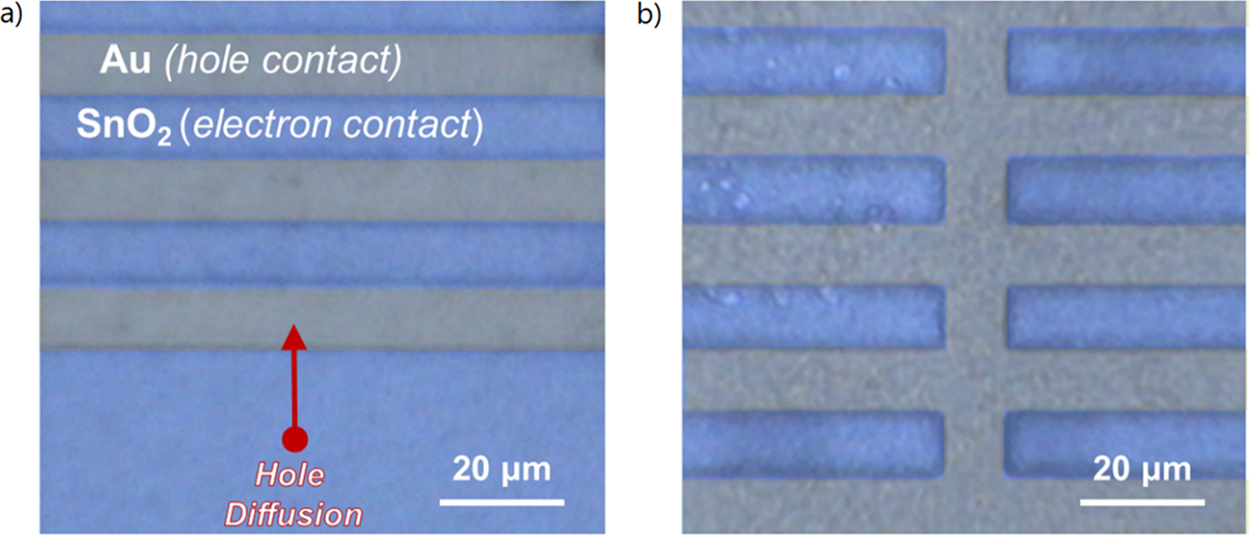
False-color optical micrograph of the quasi-interdigitated back contact device structure used for measuring lateral diffusion. Here, a pattern of an Al_2_O_3_/Au bilayer is formed on top of a continuous ITO/SnO_2_ bilayer. A thin film of perovskite is then deposited over the entire back-contact structure that remains visible under an optical microscope. Long-distance hole diffusion is measured by exciting carriers away from the last (hole-collecting) Au electrode (a) on the edge of the structure illustrated in part b.

We consider the experimental configuration illustrated in Figure 2b, measured on the quasi-interdigitated back-contact (QIBC) structure shown in Figure 6. The QIBC configuration is particularly useful for the measurement of carrier diffusion, as upon local excitation of an electron–hole pair over either electrode, one carrier will be immediately extracted while the other will need to diffuse laterally toward its respective electrode over a distance much larger than the width of the excitation spot. Such measurements have been demonstrated in the past by Tainter et al.^23^ It has also been shown by Lamboll et al. that it is valid to treat diffusion as one-dimensional in photocurrent measurements on the QIBC device structure.^35^

Here, a sample with a back contacted geometry is illuminated from above by a pulsed laser with a variable excitation frequency. Charges are generated in a spot of thickness δ at a distance *d* from the collecting contact, as illustrated in Figure 2b and Figure 6a. The photogenerated electrons are immediately extracted through the underlying SnO_2_ layer, while the holes need to diffuse laterally over a distance *d* to reach the nearest gold electrode. The experimental setup is described in more detail in the Supporting Information. Here, we provide a theoretical model for this experiment. We solve eq 1 with the generation profile *G*(*x*) = β^–1^Φ, where β is an absorption coefficient, only for *d* ≤ *x* ≤ *d* + δ, and *G*(*x*) = 0 elsewhere. The boundary condition is *n*(*x* = 0) = 0. This problem is solved in the SI and the result is
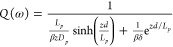
13

**Figure 7:**
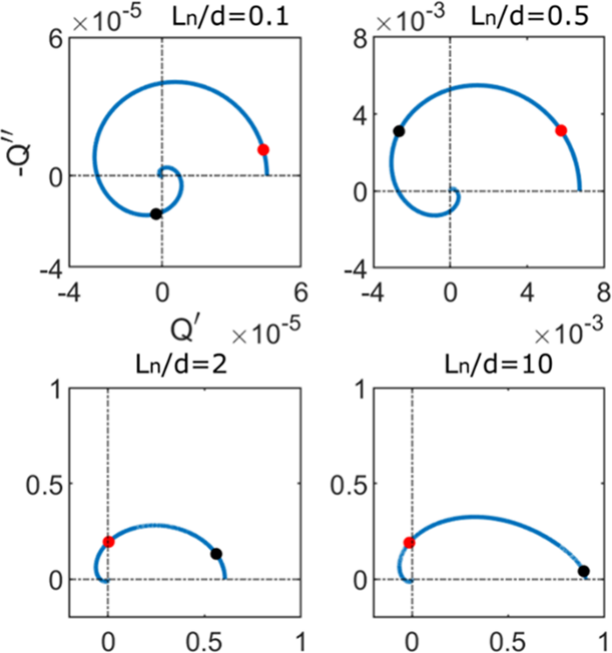
Complex plane IMPS function plots for different diffusion lengths. The absorption depth has been fixed to β^–1^ = 0.01 μm and the diffusion length also, *L*_*n*_ = 1 μm. The cell thickness is considered to be the same as the absorption depth. The characteristic time constants are defined in Figure 3.

The IMPS function is plotted for different cases of the diffusion length in Figure 7. Loops appear whatever the chosen diffusion length is. In the low diffusion length case, a shell shape is obtained. A small value of the EQE is obtained as the carriers are mainly recombined in the material. The IMPS function crosses over to the second quadrant with a complex exponential dependence like the dependences of the first method. When we increase the diffusion length, the EQE increases as the carriers easily diffuse in the sample. The shapes become longer than higher, but the function still leaves the first quadrant spiraling to the origin as in the other cases.

For the analysis of the data, the diffusion transfer function is complemented by other standard elements:^36^*R*_s_, series resistance; *R*_HF_, high-frequency resistance; *C*_g_, geometrical capacitance. The overall transfer function can then be written as in eq 11 with the attenuation factor:
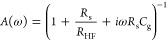
14

**Figure 8:**
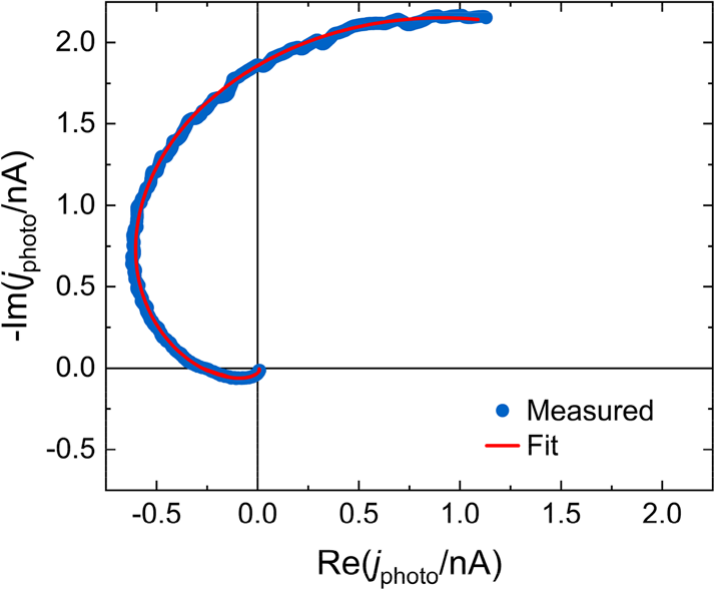
IMPS data (point) and fit (line) for a back contacted perovskite cell excited by a ∼1 μm wide pulsed laser illumination spot 10 μm away from the nearest hole collecting electrode.

The measured IMPS spectrum from the configuration in Figure 2b exciting 10 μm away from the collecting electrode is shown in Figure 8, along with a fit to the product of eqs 13 and 14. Importantly, in the regime of excitation far away from the collecting electrode and *L*_*p*_ ≈ *d*, we can again clearly observe an NHF loop indicating a strong diffusive contribution to the IMPS spectrum. The fitting details are described in the Supporting Information. From the fit, we extract *L*_*p*_ = 11 μm, τ_*p*_ = 1.6 μs, and a corresponding *D*_*p*_ = 0.76 cm^2^/s. These values are once again consistent with those reported previously in literature, and the improvement in diffusivity can be attributed to the use of a triple cation, mixed halide perovskite in the back contacted configuration as opposed to MAPbI_3_ in the sandwich structure. In particular, the correspondent room-temperature hole mobility μ_*p*_ = 30 cm^2^ V^–1^ s^–1^ measured here shows good correspondence to the combined electron and hole mobility (μ = μ_*p*_ + μ_*e*_) obtained for MAPbI_3_ polycrystalline thin films by THz spectroscopy, which also measures in-plane movement of charge carriers, where μ = 33 cm^2^ V^–1^ s^–1^.^4^ The hole mobility being higher by roughly a factor of 2 for the measurement in this study can once again be attributed to the triple cation mixed halide perovskite composition used instead of MAPbI_3_.

Now that we have completed the description of measurement and data analysis let us discuss the significance for a better understanding of halide perovskite solar cells. In Figures 5 and 8, we have shown the measured data for the range of frequencies that obey the diffusion model. As mentioned, the data are affected by some additional electrical elements *R*_s_, *R*_HF_, and *C*_g_. These elements form part of a wider equivalent circuit that includes additional elements that describe also the low frequency data. The equivalent circuit for perovskite solar cells including the required elements has been discussed in other publications,^12,16,36^ and a full spectrum showing the low frequency features is shown in Figures SI.3 and SI.4. The remarkable fact is that the diffusion part of the spectra in Figure 5 is nicely separated from the low frequency arcs that relate to surface recombination and ionic polarization. In other measurement methods for the diffusion parameters in a full device, for example, the SCLC, it is unavoidable that measured dc current is influenced by the state of the contacts (especially by the application of large voltages).^5^ The high frequency IMPS method has the unique feature that it is applied in an operating device but it is not strongly affected by the conditions of contacts. Nevertheless, the overall polarization can form an electrical field and charge extraction conditions and produce modifications that alter the IMPS measurement. The method is then a powerful tool to investigate in situ the mechanisms of transport and recombination. An exploration of these more complex conditions is left for future investigations.

In summary, we have associated a negative loop spiraling to the origin of the IMPS transfer function at high frequencies, to the generation of electronic carriers in a thin region and the subsequent transport across the sample thickness to the collecting contact. This spectrum occurs under the conditions that (1) the absorption length is much shorter than the cell thickness and (2) the diffusion length is longer than the absorption distance. For an interdigitated cell with planar back contacts the illumination of a spot produces the *Q*-loops in all cases. We showed that the application of diffusion-recombination models enables a quantitative determination of the carrier diffusion coefficient and lifetime. This is the first consistent determination of electron diffusion by the small amplitude spectral method, since these features have not been obtained in impedance spectroscopy and emerge in intensity modulated photocurrent spectroscopy.

## Supporting Information Available

Calculation of IMPS function for sandwich configuration and for lateral transport, fitting of experimental data, spectral shapes of limiting functions, RC attenuation, full spectral shape, material preparation methods (PDF)
