# Supplementary material for: Extracting in Situ Charge Carrier Diffusion Parameters in Perovskite Solar Cells with Light Modulated Techniques
Source: ACS Energy Lett. 2021 May 24;6(6):2248–55. doi: 10.1021/acsenergylett.1c00871 (PMC8576830; doi:10.1021/acsenergylett.1c00871)
Supplement: Supplementary file 1 — nz1c00871_si_001.pdf [file nz1c00871_si_001.pdf]

Supporting Information

Extracting *In-Situ* Charge Carrier Diffusion Parameters in Perovskite Solar Cells with Light Modulated Techniques

Agustín Bou[a], Haralds Āboliņš,[b] Arjun Ashoka,[b] Héctor Cruanyes,[a] Antonio Guerrero,[a] Felix Deschler,[c]* Juan Bisquert[a]*

[a]Institute of Advanced Materials (INAM), Universitat Jaume I, Avda. Sos Baynat sn, 12006 Castelló, Spain

[b]Cavendish Laboratory, University of Cambridge, J.J. Thomson Avenue, CB3 0HE, Cambridge, United Kingdom

[c]Walter-Schottky-Institute, Physics Department, Technical University Munich, Am Coulombwall 4, Garching bei München, Germany

IMPS Function for sandwich solar cell configuration (Fig. 2a)

Carrier generation in the right side of the cell with an exponential profile has the expression

$$\tilde{G}(x,t) = \alpha\tilde{\Phi}(t)\exp\left(\alpha(x-d)\right) \quad \text{(A1)}$$

where $\tilde{\Phi}(t) = \tilde{\Phi}_0 \exp\left(i\omega t\right)$ is the small ac perturbation of the incident photon flux. The excess electron density has the form

$$\tilde{n}(x,t) = u(x,\omega)\exp\left(i\omega t\right) \quad \text{(A2)}$$

Equation 1 is reduced to the following expression

$$\frac{\partial^2 u}{\partial u^2} = \frac{z(\omega)^2}{L_n^2}u - \alpha\frac{\tilde{\Phi}_0}{D_n}\exp\left(\alpha(x-d)\right) \quad \text{(A3)}$$

with $z(\omega) = \left(1 + i\omega\tau_n\right)^{1/2}$. The solution is

$$u(x,\omega) = A\exp\left(z\frac{x}{L_n}\right) + B\exp\left(-z\frac{x}{L_n}\right) + C\exp\left(+\alpha x\right) \quad \text{(A4)}$$

as it has been previously shown in literature.[1] A,B and C are constants that can be calculated with the use of the boundary conditions:

$$A + B = -C \quad \text{(A5)}$$

$$A\exp\left(z\frac{d}{L_n}\right) - B\exp\left(-z\frac{d}{L_n}\right) = -C\frac{\alpha L_n}{z}\exp\left(\alpha d\right) \quad \text{(A6)}$$

Therefore

$$A = -C\frac{z\exp\left(-z\frac{d}{L_n}\right) + \alpha L_n\exp\left(\alpha d\right)}{z\left[\exp\left(z\frac{d}{L_n}\right) + \exp\left(-z\frac{d}{L_n}\right)\right]} \quad \text{(A7)}$$

$$B = C\frac{-z\exp\left(z\frac{d}{L_n}\right) + \alpha L_n\exp\left(\alpha d\right)}{z\left[\exp\left(z\frac{d}{L_n}\right) + \exp\left(-z\frac{d}{L_n}\right)\right]} \quad \text{(A8)}$$

The C constant is obtained introducing the solution into the differential equation.

$$C = \frac{\alpha L_n^2(\tilde{\Phi}_0/D_n)}{z^2 - \alpha^2 L_n^2} \exp\left(-\alpha d\right) \quad \text{(A9)}$$

The IMPS transfer function is obtained with

$$\tilde{j}_e(x = 0) = +qD_n \frac{\partial u}{\partial x} \quad \text{(3)}$$

$$Q = \frac{\tilde{j}_e}{q\tilde{\Phi}} \quad \text{(4)}$$

The excess electron density can be obtained by taking real part of the Equation A4.

IMPS Function for lateral transport configuration (Fig. 2b)

Similarly to the problem of the parallel contacts, we use Equation 1 for holes, with carrier generation in this case defined by:

$$\tilde{G}(x) = \begin{cases} 0 & 0 < x \leq d \\ \beta\tilde{\Phi} & d < x < d + \delta \\ 0 & d + \delta \leq x < +\infty \end{cases} \quad \text{(A10)}$$

Then, we have three separated regions (1, 2, 3 from right to left). To calculate the IMPS transfer function, we need to know the extracted current at the collecting point $x = 0$,

$$\tilde{j}_p(0) = qD_p \frac{\partial \tilde{p}}{\partial x}\Big|_{x=0} \quad \text{(A11)}$$

The small perturbation transform of equation 1 for regions 1 and 3 gives

$$s\tilde{p} = D_p \frac{\partial^2 \tilde{p}}{\partial x^2} - \frac{\tilde{p}}{\tau_p} \quad \text{(A12)}$$

The solution for excess density of holes in region 1 is:

$$\tilde{p}(x) = Me^{x/L} + Ne^{-x/L} \quad \text{(A13)}$$

Given that the boundary condition at the collecting contact is $p(0) = 0$, this gives $N = -M$, and therefore

$$\tilde{p}(x) = M(e^{x/L} - e^{-x/L}) \quad \text{(A14)}$$

Substituting the value of $\tilde{p}(x)$ in equation A14 we get

$$L = \frac{L_p}{\sqrt{1 + s\tau_p}} = \frac{L_p}{z} \quad \text{(A15)}$$

Similarly, the solution for excess density of holes in region 3 is:

$$\tilde{p}(x) = Re^{x/L} + Se^{-x/L} \quad \text{(A16)}$$

Here, as a boundary condition, we apply $\tilde{p}(+\infty) = 0$, which gives $R = 0$, and the excess hole density is

$$\tilde{p}(x) = Se^{-x/L} \quad \text{(A17)}$$

Assuming uniform density at the generation spot, $\tilde{p}(d) = \tilde{p}(d + \delta)$, we get

$$S = \left(e^{d/L} - e^{-d/L}\right) e^{\frac{d+\delta}{L}} M \tag{A18}$$

And solving small perturbation transform of equation 1 in the illuminated region 2, we get:

$$M = \frac{\beta\delta}{2\left(s\delta \sinh\left(\frac{dz}{L_p}\right) + \frac{1}{\beta\delta} e^{\frac{dz}{L_p}}\right)} \tilde{\Phi} \tag{A19}$$

Which gives the extracted current:

$$\tilde{j}_p(0) = qD_p \frac{\partial \tilde{p}}{\partial x}\bigg|_{x=0} = 2q\frac{zD_p}{L_p} M = \frac{zD_p}{L_p} \frac{\beta\delta}{s\delta \sinh\left(\frac{dz}{L_p}\right) + \frac{1}{\beta\delta} e^{\frac{dz}{L_p}}} q\tilde{\Phi} \tag{A20}$$

In this case, the definition of the IMPS transfer function Q will be slightly different from Equation 4, since the illuminated and collecting areas are not the same. Therefore, we will define it as:

$$Q = \frac{a\,\tilde{j}_e}{\delta q \tilde{\Phi}} = k\frac{\tilde{j}_e}{q\tilde{\Phi}} \tag{A21}$$

Where a and δ are the collecting and illumination transversal length, respectively. Since k is an experimental factor that depends on the configuration, we calculate the IMPS transfer function for $k = 1$ in equation (16). In practice a normalizing factor in Q can be used as a fitting parameter, since obtaining the EQE is not a priority in this configuration.

Fitting the IMPS Function for sandwich solar cell configuration (Fig 2a)

The RC attenuation $A(\omega)$ describes that the diffusion is accompanied by a larger equivalent circuit.

$$Q_{meas}(\omega) = A(\omega)Q(\omega) \tag{15}$$

For the sandwich solar cell configuration, the series resistance and the geometrical capacitance can be measured via Impedance Spectroscopy. The absorbance length is obtained from bibliographic sources.[2] The list of fitting parameters, used for the elaboration of Fig. (5), is indicated in Table S1.

Table S1. Parameters used in IMPS fitting function in Fig. (5) for sandwich solar cell configuration.

Light	α^{-1} (nm)	d (µm)	R_S (Ω cm^2)	C_g (nF/cm^2)
Blue	40	5.3	10	3.6
Red	140			

Fitting the IMPS Function for lateral transport configuration (Fig. 2b)

In addition to attenuation, two more fitting parameters had to be introduced. Firstly, since the photocurrent amplitude for IMPS in the lateral transport configuration was measured in arbitrary units, a normalization factor N had to be introduced such that

$$Q_{meas}(\omega) = N \times A(\omega)\, Q(\omega). \quad \text{(A21)}$$

Secondly, in order to account for possible anomalous diffusions effects[3] a power law in frequency is introduced such that

$$Q_{meas}(i\omega) = Q_{meas}[(i\omega)^p]. \quad \text{(A22)}$$

The full list of fitting parameters extracted of Fig. 8 can be found in Table S2 below.

Table S2. Parameters extracted in IMPS fitting function in Fig. (8) for the lateral transport configuration.

N	p	R_S (Ω cm^2)	R_3 (Ω cm^2)	C_g (μF/cm^2)	L_p (μm)	D_p (cm^2/s)
3.4×10^5	1.26	19	3.8	1.1	11	0.76

Spectral Shapes of the Limiting Functions

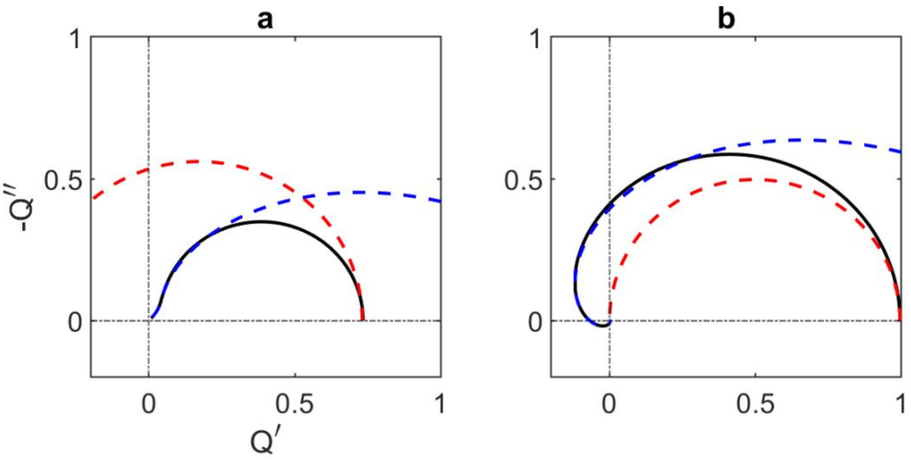

Figure SI.1. Complex plane plots of the IMPS transfer function (black line) for $L_n = 10d$ and different absorption lengths (a) $\alpha d = 4/3$ and (b) $\alpha d = 10$. The limiting functions are represented in the high frequency limit (blue dashed line) and in the low frequency limit (red dashed line). No RC attenuation is considered.

To better appreciate the analytical shape of the function of interest we present some approximations that can be obtained when the diffusion length is longer that the cell

thickness ($L_n < d$) and the light is completely absorbed in a short region. The high frequency limit is

$$Q(\omega) \approx 2\frac{\exp\left(-\frac{d}{L_n}\sqrt{i\omega\tau_n}\right) - \frac{1}{2}\exp\left(-\alpha d\right)\left(1 + \frac{1}{L_n\alpha}\sqrt{i\omega\tau_n}\right)}{\left(1 - i\omega\tau\frac{1}{L_n^2\alpha^2}\right)} \quad (1)$$

It represents a positive arc as it is shown in Figure SI.1a. The low frequency limit is

$$Q(\omega) \approx 2\frac{1 - \exp\left(-\alpha d\right)\left(1 + \frac{d}{\alpha L_n^2}(1 + i\omega\tau_n)\right)}{\left(2 + \frac{d^2}{L_n^2} - \frac{d^2}{L_n^4\alpha^2}\right) + i\omega\tau\left(\frac{d^2}{L_n^2} + \frac{2d^2}{L_n^4\alpha^2}\right)} \quad (2)$$

This function forms a semicircle that starts in the negative axis. As the absorption length becomes shorter, $\alpha d \gg 1$ the terms with the negative exponential become irrelevant. The limiting functions for high and low frequency turn into the following expressions, respectively:

$$Q(\omega) \approx \frac{2}{\left(1 - i\omega\tau_n\frac{1}{L_n^2\alpha^2}\right)}\exp\left(-\frac{d}{L_n}\sqrt{i\omega\tau_n}\right) \quad (3)$$

$$Q(\omega) \approx \frac{2}{\left(2 + \frac{d^2}{L_n^2} - \frac{d^2}{L_n^4\alpha^2}\right) + i\omega\tau_n\left(\frac{d^2}{L_n^2} + \frac{2d^2}{L_n^4\alpha^2}\right)} \quad (4)$$

The spectral dependences of these functions are shown in Figure SI.1b. The complex exponential function that depends on $\sqrt{i\omega\tau_n}$ is the one that loops and spirals into the negative Q' axis.

The RC attenuation effect

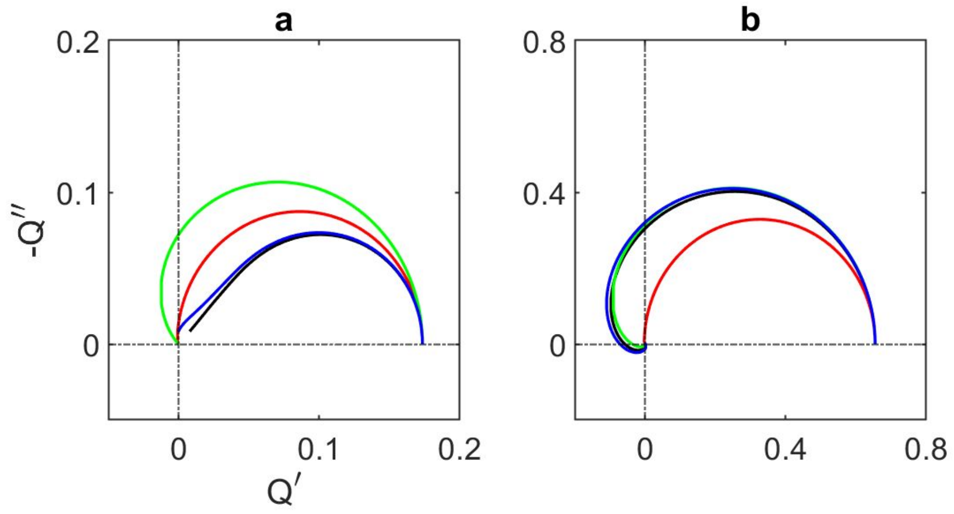

Figure SI.2. Complex plane plots of the simulated IMPS transfer function considering RC attenuation in different absorption and diffusion lengths (Eq.12,13). The black line is the theoretical IMPS transfer function ($RC = 0$), the red line is for a high RC factor ($RC =$

$10^2\tau_n$), the green line is for an intermediate value ($RC = \tau_n$) and the blue line represents the low limit value ($RC = 10^{-2}\tau_n$). (a) Cell distance $d = 10^{-6}$µm, diffusion coefficient of $D = 0.001\ \mathrm{cm^2/s}$, absorption length of $\alpha d = 4/3$ and recombination lifetime $\tau_n = 1$ µs. (b) absorption length of $\alpha d = 10$ and lifetime 10 µs.

Measured IMPS Function for all the whole measured frequency range

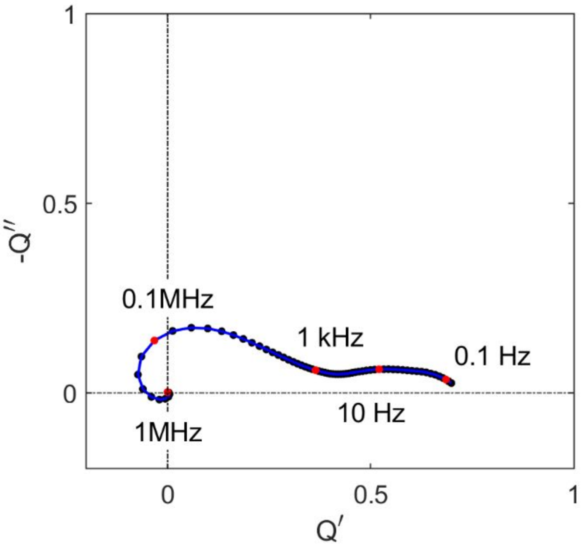

Figure SI.3. Complex plane plot of the measured IMPS transfer function as fitted in Fig. 5 for blue light. Measurements are shown for a wider frequency range down to 0.1 Hz . Red points denote specific frequencies as written.

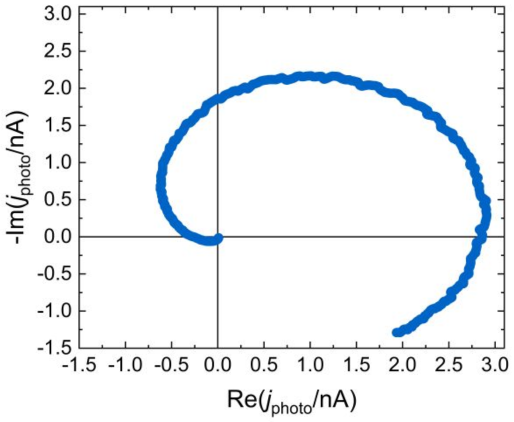

Figure SI.4. Complex plane plot of the measured IMPS transfer function as fitted in Fig. 8. Measurements are shown for a wider frequency range down to 5 Hz.

Methods

Interdigitated back contact solar cell fabrication

ITO was deposited by sputter coating 150 nm on SiO2 (10 μm thick) on Si chips. SnO_2 was deposited by diluting a colloidal dispersion (15% in H_2O, purchased form Alfa Aesar) to 2% and spincoating for 30 s at 3000 rpm and baking on a hot plate at 150 °C for 30 min. An insulating layer of AlO_x (150nm) was deposited by atomic layer deposition on the chips. The quasi-interdigitated electrode pattern was written using e-beam lithography. After development, Cr/Au (2/70 nm) was deposited by thermal evaporation followed by lift-off. The chips were then placed in the TMAH-based AZ726 MIF developer, which etched the AlO_x not covered by the electrodes, leaving the Au electrodes electrically insulated whilst exposing the SnO_2

The organic cations (FAI and MABr) were purchased from GreatCell solar, the lead compounds (PbI_2 and $PbBr_2$) were purchased from TCI and the CsI was purchased from Sigma Aldrich. The mixed cation perovskite solution was prepared by mixing FAI (1 M), MABr (0.2 M), PbI_2 (1.1 M) and $PbBr_2$ (0.2 M) in anhydrous DMF:DMSO 4:1 (v:v). After that, 5% of 1.5 M CsI was added to the prepared mixed cation perovskite solution to achieve triple cation perovskite solution. Before depositing perovskite the patterned electrode substrate was cleaned with UV-ozone treatment for 15 min. The film perovskite was spin-coated with a two-step program at 1000 and 6000 rpm for 10s and 20s respectively. During the second step, 100 μl of antisolvent anhydrous chlorobenzene was dropped on the substrate, 5 second before the end of program. After spin-coating, the substrate was annealed on a hotplate at 100 °C for 1h. The spin-coating and annealing steps were performed in a N2-filled glovebox.

IMPS measurement in lateral transport configuration

A HP 8116A 50 MHz programmable function generator was used to generate two synchronous signals - one with a 50% duty cycle which allowed for comfortable reference signal phase locking with the lock-in amplifier and a second TTL signal with an on-time of < 40ns and with voltage limits set to appropriately trigger the laser system. The laser system used was a Pico Quant picosecond pulsed diode laser driver PDL 800-D with a LDH405 pulsed laser diode head (405 nm excitation wavelength, pulse length of 20ps) to appropriately excite above the bandgap of perovskite being studied. A Zurich Instruments HF2LI 50 MHz Lock-in Amplifier was used for the lock-in amplification of the current signal from the device and the HF2 LabOne software was used for frequency, lock-in signal and phase acquisition. The stage on which the sample was placed, and the confocal microscope was part of a WITec alpha 300 s setup. The incident power over the range of repetition rates studied was between 7nW and 5uW and the photocurrent extracted had an expected linear dependence of the average power on frequency. The maximum fluence

studied (at 2MHz) by the air objective focused 1.5um laser spot was about 300 W/cm^2. The excitation frequency was swept between 20 kHz to 500 kHz.

References

1. Dloczik, L.; Ileperuma, O.; Lauerman, I.; Peter, L. M.; Ponomarev, E. A.; Redmond, G.; Shaw, N. J.; Uhlendorf, I., Dynamic Response of Dye-Sensitized Nanocrystalline Solar Cells: Characterization by Intensity-Modulated Photocurrent Spectroscopy. *The Journal of Physical Chemistry B* **1997**, *101*, 10281-10289.
2. Sun, S.; Salim, T.; Mathews, N.; Duchamp, M.; Boothroyd, C.; Xing, G.; Sum, T. C.; Lam, Y. M., The Origin of High Efficiency in Low-Temperature Solution-Processable Bilayer Organometal Halide Hybrid Solar Cells. *Energy & Environmental Science* **2014**, *7*, 399-407.
3. Bisquert, J.; Compte, A., Theory of the Electrochemical Impedance of Anomalous Diffusion. *Journal of Electroanalytical Chemistry* **2001**, *499*, 112-120.
